# Are Findings of Key Insect Metrics Generalizable Across Different Taxa in Malaise Trap Samples?

**DOI:** 10.1002/ece3.72006

**Published:** 2025-08-15

**Authors:** Nicole Remmel, Julian Enss, Peter Haase, James S. Sinclair

**Affiliations:** ^1^ Department of River Ecology and Conservation Senckenberg Research Institute and Natural History Museum Frankfurt Gelnhausen Germany; ^2^ Faculty of Biology University of Duisburg‐Essen Essen Germany; ^3^ Centre for Water and Environmental Research University of Duisburg‐Essen Essen Germany

**Keywords:** biomass, community, insect, malaise trap, pollinator

## Abstract

Recent reports of insect declines and drivers thereof are often based on total biomass from Malaise traps. However, it remains unclear whether these changes reflect shifts in other community metrics (e.g., total abundance) and important taxa, such as key pollinators. To address this question, we collected Malaise trap samples from four different habitats (forest, urban, agriculture and open land) and four seasons (early spring, late spring, midsummer and early autumn) during 2019–2021. We measured the total biomass of each sample, then morphologically identified the insects in each sample, comprising 533,128 total individuals. We determined whether changes in total biomass reflected changes in total community abundance, and whether community relationships to habitat characteristics of land cover, weather/climate, and flowering plants were the same between the most common insects (represented by 15 different taxonomic groups) versus relatively fewer focal pollinators, specifically bees, butterflies, and hoverflies. Biomass was generally related to abundance, except in a small subset of communities comprising more larger‐bodied taxa. Additionally, both overall community composition and pollinator composition were explained by the same weather and climate variables and the same habitat characteristics. However, pollinator relationships to habitat were likely driven by different mechanisms, specifically covarying changes in flowering plants. Our results suggest that patterns in Malaise trap biomass, and the relationships of prominent taxa to habitat characteristics, could be used to infer similar changes in other important community metrics and taxa, including pollinators. However, some insects responded to shifts in habitat characteristics for different underlying reasons, indicating the need for caution when using such inferences to inform conservation and management to ensure the correct mechanisms are being addressed.

## Graphical abstract

Malaise traps are increasingly used for global insect monitoring. However, it remains unclear whether patterns in total insect biomass from these traps reflect only changes in dominant taxa, or whether they reflect changes in other key community metrics and taxonomic groups. Using traps placed in four different habitats and counting all collected insects, we found that differences in total biomass generally reflected differences in total community abundance, and that habitat patterns in dominant taxa reflected similar patterns in a minority of key pollinators.

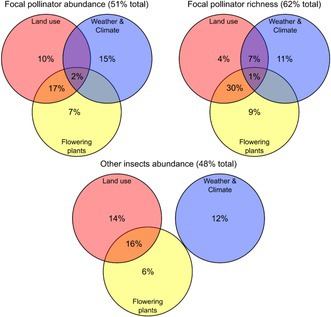

## 1 Introduction

Reports of declines in flying insects during recent decades (Shortall et al. 2009; Hallmann et al. 2017; Lister and Garcia 2018) suggest substantial losses of insect biodiversity and key ecological functions, such as pollination or decomposition (Cardoso et al. 2020; Powney et al. 2019; Ghisbain et al. 2023, 2024; Dangles et al. 2011). However, questions remain about the broader representativeness of these studies, and the drivers of reported trends. Many insect monitoring programs, including those finding substantial declines, report trends only in total biomass from Malaise trap catches (e.g., Welti et al. 2022; Hallmann et al. 2021, 2017; Svenningsen et al. 2022). This method is cost‐effective and samples a broad range of flying insects, and Malaise trap biomass can be a useful proxy for insect richness (e.g., Sinclair et al. 2024). However, biomass loss does not necessarily equate to losses in other important metrics, such as abundance (i.e., the total number of individuals in a community). A potential disconnect between biomass and abundance could occur if changes in Malaise trap biomass are primarily driven by certain taxa. For example, Malaise trap catches tend to be dominated by Diptera (Karlsson et al. 2020; Srivathsan et al. 2023); thus, declines in total biomass may primarily reflect losses of Diptera abundance while being a poor indicator of change in other taxonomic groups (Busse et al. 2022; Redlich et al. 2021). Similarly, biomass loss may primarily reflect declines only in the abundance of larger‐bodied individuals (Shortall et al. 2009; Vereecken et al. 2021) and therefore may not reflect changes in the abundance of other, smaller‐bodied taxa. These issues highlight the need for a better understanding of the relationship between Malaise trap biomass and community abundance, and how this relationship is affected by the body size distribution of the community.

Furthermore, variation in insect biodiversity has been attributed to several different habitat characteristics, including human land use, climate change (Sánchez‐Bayo and Wyckhuys 2019; Wagner 2020; Raven and Wagner 2021), and their combined effect on plant resources (Outhwaite et al. 2020, 2022; Fenoglio et al. 2021). However, owing to difficulties in capturing and identifying all insects, the studies that underlie these conclusions often focus on either broad community metrics (e.g., total biomass or species richness from Malaise traps; Welti et al. 2022; Uhler et al. 2021) or individual taxonomic groups, particularly key pollinators such as bees, butterflies, and hoverflies (e.g., Ganuza et al. 2022; Ghisbain et al. 2024; Powney et al. 2019). This focus makes it difficult to determine whether different taxa are responding to the same drivers, given that responses can vary widely among taxonomic groups, with some declining whereas others remain stable or even increase (Outhwaite et al. 2020; Saunders et al. 2020; Crossley et al. 2020). For example, as discussed above, Malaise traps are effective at capturing Diptera, which tend to dominate both total biomass and total species diversity (Karlsson et al. 2020; Buchner et al. 2025). Studies identifying habitat‐related differences in total biomass or richness may therefore be primarily identifying drivers of Diptera, and these drivers may not hold the same importance for other taxonomic groups. Similarly, other common focal taxonomic groups, such as bee, butterfly, and hoverfly pollinators, often comprise a low proportion of total Malaise trap biomass (Karlsson et al. 2020), partly because Malaise traps are less effective at capturing such taxa (e.g., bees; Krahner et al. 2021; Packer and Darla‐West 2021). Drivers identified for a small number of less effectively trapped taxa may not hold the same importance for the majority of other insects. For example, pollen or nectar feeding insects like bees and butterflies (especially plant specialists or species active in early spring) can be more influenced by shifts in flowering plants compared to other taxa (Scaven and Rafferty 2013; Forrest and Thomson 2011; Rafferty and Ives 2012; Pyke et al. 2016; Gérard et al. 2020). Addressing these questions requires Malaise trap studies that examine a wide range of taxa, including pollinators and non‐pollinators, and that identify the relative importance of multiple drivers for different groups.

**FIGURE 1:**
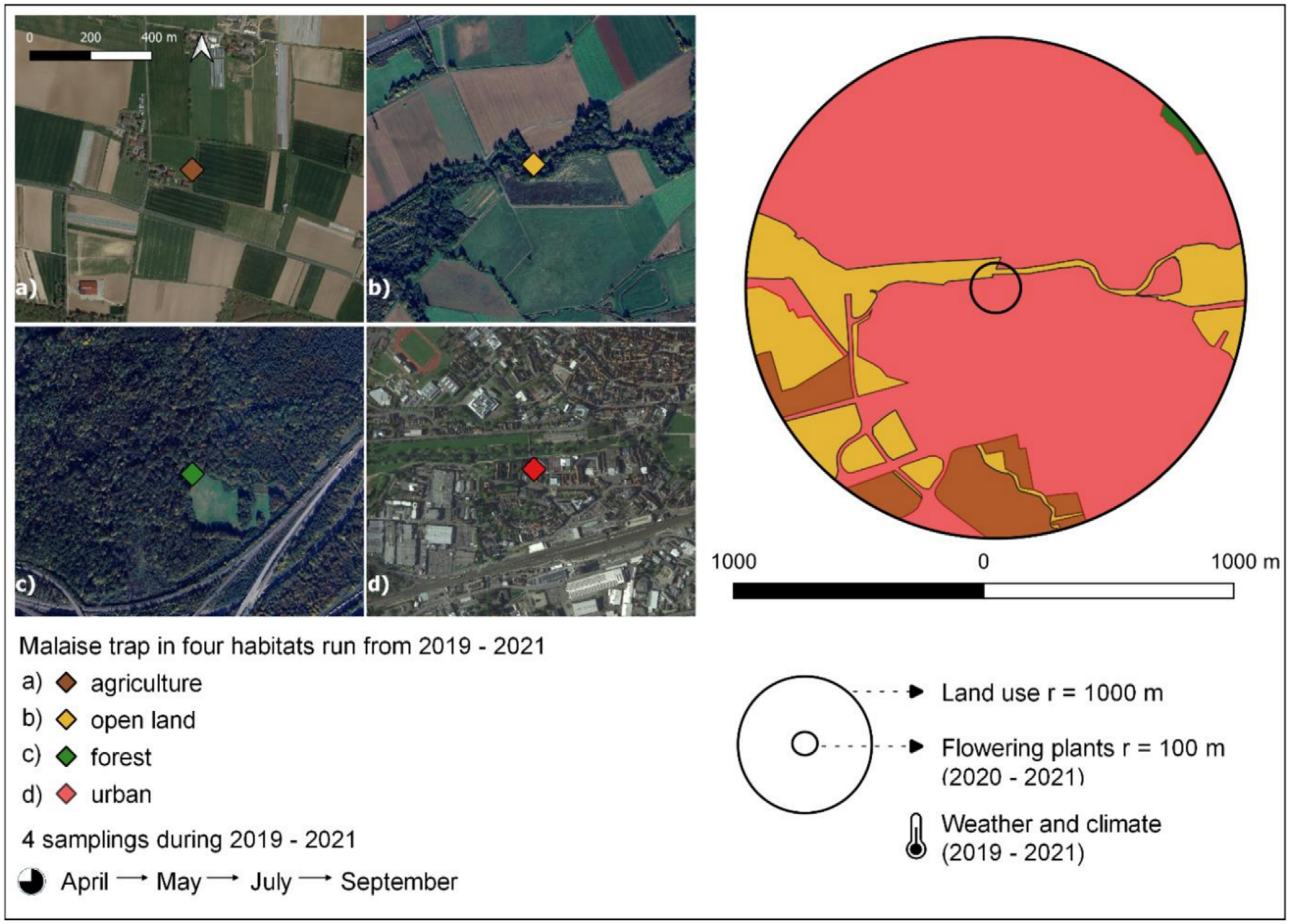
Sampling design using Malaise traps. Left side shows trap locations and sampling seasons in four different habitat types of: (a) agricultural (N 50.17989, E 8.95835; 121 m a.s.l.), (b) open land (N 50.18603, E 9.09684; 122 m a.s.l.), (c) forest (N 50.14157, E 8.98389; 115 m a.s.l.) and (d) urban (N 50.19838, E 9.18597; 131 m a.s.l.) in the Rhine‐Main‐Observatory in Germany. Right side shows the radii used to collect data on land use and flowering plants.

Here, we addressed the above knowledge gaps using insects collected from Malaise traps in a Long‐Term Ecological Research (LTER) site in Germany. These traps encompass four different habitat types—forest, open land, urban, and agricultural (Figure 1) – and provide differences in land cover, weather, climate, and flowering plant composition. Our first objective was to quantify the relationship between total wet weight biomass from Malaise trap samples and total community abundance of taxa from morphological identification. For our second objective, we quantified the proportion of the community belonging to: (1) ‘focal pollinators’, encompassing bees, butterflies, and hoverflies (representing commonly investigated pollinator groups); and (2) ‘other insects’ comprised of 16 other taxonomic groups. We then determined whether variation in focal pollinator composition among habitats versus that of other insects was related to the same differences in land cover, weather/climate, and the composition of flowering plants. We expected that the relationship between biomass and abundance would depend on the body size composition of the community, with a potentially weaker relationship in communities comprised of more larger‐bodied taxa. We also expected that differences in flowering plants would explain more variation among focal pollinators compared to the other insect groups. Our results may help generalize relationships between the multiple components of Malaise trap insect communities, increasing our still insufficient understanding of the methodology and the biodiversity patterns it is used to identify.

## 2 Methods

### 2.1 Research Area and Sampling Sites

The research area is located in the Rhine‐Main‐Observatory (RMO; https://deims.org/9f9ba137‐342d‐4813‐ae58‐a60911c3abc1), a Long‐Term Ecological Research (LTER) site from central Germany comprising the entire catchment of the Kinzig river (1058 km^2^; Haase et al. 2016; Mirtl et al. 2018) and being part of the German Malaise Trap Program. This program was established in 2019 and comprises 75 locations across Germany used to determine the biomass and species composition of flying insect communities (Welti et al. 2022). In our study, we investigated samples of flying insects caught in four Townes‐type Malaise traps (black roofs and 1.2 m^2^ openings on both sites; Uhler et al. 2022) in four different habitat types at the RMO (Figure 1). Distances between the four sampling sites were between 6.5 and 16.3 km, which we consider as providing sufficient independence among communities given that Malaise traps just tens or hundreds of meters apart capture substantially different taxa (e.g., Steinke et al. 2021; Chimeno et al. 2023). Samples were collected after traps were out for 14 days, with collections occurring in early spring, late spring, mid‐summer, and early autumn during 2019, 2020, and 2021 to account for seasonal community changes. Insects were captured in bottles filled with 80% denatured ethanol (1% methyl ethyl ketone). After field collection, biomass was wet weighed after filtering alcohol with a stainless steel sieve (0.8 mm mesh width) following the procedure in Hallmann et al. (2017) and was then re‐preserved in 96% denatured ethanol for future morphological identification. A total of 48 samples were collected from the combination of 3 years, 4 sampling dates per year, and 4 sampling sites (Figure 1).

### 2.2 Morphological Identification

Collected insects were counted and divided into two groups. The first group included what we hereafter refer to as ‘focal pollinators’, which encompassed: bees (i.e., Apiformes), butterflies (Lepidoptera‐Hesperiidae, Lycaenidae, Nymphalidae, Pieridae), and hoverflies (Syrphidae). The second group included 15 different taxonomic groups, hereafter referred to as ‘other insects’, which encompassed: (1) Blattodea (cockroaches); (2) Coleoptera (beetles); (3) Dermaptera (earwigs); (4) non‐Syrphidae Diptera (other flies); (5) Ephemeroptera (mayflies); (6) Hemiptera (Auchenorrhyncha; leafhoppers, treehoppers, spittlebugs, cicadas and planthoppers); (7) Hemiptera (Heteroptera; true bugs); (8) non‐Apiformes Hymenoptera; (9) Lepidoptera (moths); (10) Mecoptera (scorpionflies); (11) Neuroptera (net‐winged insects); (12) Odonata (dragon‐ and damselflies); (13) Orthoptera (grasshoppers, crickets and their allies); (14) Plecoptera (stoneflies); and (15) Trichoptera (caddisflies). We also counted a 16th group encompassing: (i) all other taxa that are captured by Malaise traps but are not insects; (ii) those that only occurred in a single sample; and (iii) taxa that are difficult to morphologically identify, all of which typically only comprised about 1%–3% of total community abundance. These taxa were included in our total abundance counts but not our community analyses (Table 1) and encompassed: Arachnida (spiders), Collembola (springtails), Isopoda (woodlice), Raphidioptera (snake flies), Strepsiptera (twisted‐wing insects), Megaloptera (alderflies), Thysanoptera (thrips), Psocoptera (booklice), and Hemiptera (Sternorrhyncha; plant lice). Focal pollinators were identified to species level using keys for German insects (Table S1) owing to the ready availability of taxonomists for this group. Given difficulties in representing compositional relationships for hundreds of species of focal pollinators, we present these results at the family level. The other insects were identified to the above listed order or suborder level.

**TABLE 1:** Overview of taxonomic groups and their consideration in analyses.

Section	Analysis	Variable
Abundance to biomass relationship	Total abundance ~ total biomass * proportion of larger‐bodied taxa^a^	Total (summed) abundance of focal pollinators^b^ and all other insects (groups 1–16)
		Total wet weight biomass
		Proportion of larger‐bodied taxa: bees, butterflies, and moths
	Hoverfly abundance ~ total biomass	Total abundance of hoverflies
	Bee abundance ~ total biomass	Total (summed) abundance of all bee families
Community composition to environmental drivers relationship	Focal pollinator^b^ abundance ~ drivers	Abundance of each focal pollinator^b^ family
		Drivers: weather & climate (Temperature, precipitation, and humidity)
		Drivers: land use (agricultural, forest, open land, and urban)
		Drivers: Cover of each of the 20 most common flowering plant families
	Focal pollinator^b^ richness ~ drivers	Species richness of each focal pollinator^b^ family
	Other insect abundance ~ drivers	Total (summed) abundance of each other insect group (groups 1–15^c^)

^a^ Typically exhibit the largest/heaviest body sizes in our study region.

^b^ Focal pollinators = bees, hoverflies and butterflies.

^c^ Group 16 was excluded from these analyses.

### 2.3 Biomass of Hoverflies and Bees

In addition to total biomass, we also separately quantified the biomass of hoverflies and bees after morphologically identifying these taxa (Table S1). We did so to inform our examination of the biomass‐abundance relationship by obtaining data on two groups that may exhibit taxon‐specific differences in this relationship. Specifically, previous research has shown a strong relationship between total Malaise trap biomass and hoverfly abundance (Hallmann et al. 2021). In contrast, bee biodiversity may exhibit a weaker relationship (Redlich et al. 2021), partly due to their often larger body sizes (e.g., bees in our region are typically > 10 mm). We did not consider butterflies for these counts because they did not occur in many samples and, if they did, at generally low abundances.

### 2.4 Land Cover

To determine the degree to which land cover explained compositional variation among habitats, we recorded land cover information within a 1 km radius around each trap during 2020 based on biotope codes from Land Hessen (2018), which is commonly used for classifying biotope types in our study region. We also examined a 500 m radius, but found these values were similar to the 1 km values (see Data S1). We identified in the field four main land cover types of: (1) agriculture (cover of farmland, nurseries, and allotments); (2) forest (cover of deciduous and coniferous trees); (3) open land (cover of grassland and shrubland); and (4) urban (cover of urban sealed area including roads). Each site in Figure 1 was dominated by its respective land cover type (Data S1). After field mapping, the cover of each of the four land cover types was quantified by overlaying the field maps onto Google Earth satellite imagery (2024) in qGIS (v3.28 Firenze) then using the field calculator. Cover data was only collected during 2020 because the RMO exhibits little year‐to‐year land cover change, so we used the 2020 values to represent land cover for all samples.

### 2.5 Flowering Plants

To determine the influence of flowering plants, we recorded all plant species in flower, excluding grasses, mosses, and lichen, during the period of all Malaise trap samplings conducted in 2020 and 2021, but not in 2019 owing to logistical constraints. All flowering plants within a 100 m radius around each Malaise trap were recorded, which is a frequently used sampling distance in studies of plant–pollinator interactions (e.g., a 50–200 m radius is common; Forrest and Thomson 2011; Suni and Whiteley 2015; Ganuza et al. 2022; Hallmann et al. 2017; Ssymank and Doczkal 2017). Four recordings per year were conducted during 1 day within each 14‐day Malaise trap sampling period. Species identification was performed directly in the field using identification keys for German plants (Ritz et al. 2021). For each plant species in flower, abundance was quantified based on one of six abundance classes of: 1–5, 6–20, 21–200, 201–1000, 1001–5000, and 5001–15,000 individuals. Given that we identified hundreds of plant species, and we cannot statistically test relationships between these species and insect communities from just 36 samples, we instead raised all species‐level identifications to the family level and retained only the 20 most common plant families (Data S1). To quantify the abundance of each family for each sample, we summed the abundance of each observed species within each family using the mean value of the abundance class of each species.

### 2.6 Weather and Climate

To represent within‐year differences in temperature, humidity, and precipitation (i.e., weather) among sites and sampling periods, we calculated mean daily temperature, mean daily humidity, and total daily precipitation for each site across each 14‐day sampling period (Data S1). Temperature and humidity were obtained from the European Climate Assessment & Dataset gridded observational dataset (E‐OBS; 0.1 × 0.1° resolution; https://www.ecad.eu). Precipitation data was obtained from the German Weather Service (1 × 1 km resolution; https://opendata.dwd.de). To represent differences in long‐term temporal changes in temperature, humidity, and precipitation (i.e., climate change) among sites and sampling periods, we quantified the deviation in mean temperature, humidity, and precipitation for each sampling period compared to the 30‐year average for these same periods during 1989–2018. We hereafter refer to these deviations as ‘climate anomalies’. Additionally, given that variation in winter climate can also influence insects (Bale and Hayward 2010), we included the deviations between temperature, humidity, and precipitation during the winter (Nov–Mar) previous to each sampling year and the winter averages across the previous 30 years, which we hereafter refer to as ‘winter climate anomalies’. Note that the forested and agricultural sites are located in the same grid in E‐OBS, and so use the same temperature and humidity values.

### 2.7 Statistical Analyses

To quantify the relationship between biomass and abundance, we related a continuous response variable of the total abundance (individuals) of all morphologically identified taxa to a continuous predictor variable of the total wet weight biomass (g) of each sample using a generalized linear mixed model (summarized in Table 1; *n* = 48). We examined the influence of larger‐bodied taxa on this relationship by including an interaction with the continuous term for the proportion of the community comprised of butterflies, moths, and bees, which typically exhibit the largest/heaviest body sizes in our study region (Table S1; Hoffmann et al. 2025). Our expectation was that the slope of the relationship could differ for communities comprised of more larger‐bodied taxa. We also included an interaction with the quadratic term for abundance (i.e., abundance^2^) to model non‐linear relationships. We did not consider higher‐order polynomials as these would require too many interactions for a model with only 48 data points. Additionally, we included three categorical random intercept effects for sampling site, year, and season to control for non‐independence among samples collected from the same places and times. The model was performed with the glmmADMB package (Fournier et al. 2012; Skaug et al. 2016) in R (R Core Team 2023). Abundance was modeled using a Negative Binomial distribution with a log link function. Model assumptions were checked using plots of both default and scaled Pearson residuals versus fitted values, dispersion (Φ in the Results), and by visually examining the fit to the data. Significance (*p* < 0.05) of fixed terms was determined by dropping them from the model and comparing the likelihood of the reduced versus fuller models using Likelihood Ratio Tests (LRTs; Zuur et al. 2009). The explained variance of the model was determined as the marginal‐*R* ^2^ calculated using the *r.squaredGLMM* function from the MuMIn package (Bartoń 2024). We repeated the above analysis for hoverflies and bees to examine differences in the biomass‐abundance relationship for these taxonomic groups. The random effect structure was the same as above; however, we only included the continuous term for the biomass of each taxon as a fixed predictor because there was no need to test for the influence of larger‐bodied taxa for an individual taxonomic group. These models also used a Gaussian distribution with an identity link function, which provided a better fit compared to a Negative Binomial distribution.

We used a series of multivariate analyses to determine the degree to which land cover, weather/climate, and flowering plants explained variation in both focal pollinators and other insects (Table 1). For the focal pollinators, their species‐level identifications allowed us to examine relationships to both the abundance and species richness of each family. For the other insects, we only examined relationships to the abundance of each taxon. Both abundance datasets were transformed following Anderson et al. (2006) using a base of 2, specifically as *x*' = $\log_{2}$(*x*) + 1, unless *x* = 0, in which case *x*' = 0, where *x*' represents the transformed abundance and *x* represents the untransformed abundance. This transformation preserves zero values and using log base 2 ensures that all taxa contribute more equally, instead of the results being driven by the most abundant taxa. Focal pollinator richness was not transformed because the range of values was more similar across taxa. Additionally, we controlled for seasonal differences in composition by relating each community dataset to a categorical term for sampling season using linear models and extracting the residuals. This approach was necessary to focus on differences among habitats and years, rather than seasonal changes that are primarily determined by temperature.

Our first set of analyses used variation partitioning to quantify how much among‐sample variation in each of the three compositional datasets was explained by individual variation in land cover, weather/climate, and flowering plants, and to covariation among these three driver groups (e.g., the forested habitat tending to also be wetter and colder and to harbor specific plants). The same predictor datasets were used in all analyses. We removed any individual driver variables that exhibited substantial covariation with others in the same group (based on variance inflation factors > 10). All driver variables were also scaled, i.e., centered to their respective across‐sample means and divided by the standard deviation, to ensure all contributed equally to the analyses. Note these analyses only examined community and driver data for samples from 2020 and 2021 (*n* = 36) because flowering plants were only surveyed in these years. Variation partitioning was performed with the *varpart* function from the vegan package (Oksanen et al. 2022).

Our second set of analyses separately related each compositional dataset to each driver dataset using Redundancy Analysis (RDA; land cover: *n* = 48; weather/climate: *n* = 48, flowering plants: *n* = 36). This approach allowed us to examine the relationships of individual taxa to individual drivers. All response and predictor variables were transformed as detailed above for the variation partitioning analyses. RDAs were performed with the *rda* function from the vegan package.

## 3 Results

Across 48 samples, we identified 533,128 individual insects, with sample‐level total abundance ranging from 830 to 25,789 individuals. The agricultural site exhibited the highest abundances, while the lowest abundances were found at the forested site (Table 2). Total sample biomass ranged from 3.2–115.8 g, which translated to 0.23–8.3 g day^−1^ given all traps were out for 14 days.

**TABLE 2:** Overview of taxonomic groups and their total individuals across four habitat types summed across all sampling periods during 2019–2021.

Taxonomic groups	Agriculture	Forest	Open land	Urban	Total individuals 2019–2021
non‐Syrphidae Diptera	134,932	74,590	90,957	97,802	398,281
non‐Apiformes Hymenoptera	22,862	20,013	21,694	19,835	84,404
Hemiptera (Auchenorrhyncha)	3068	2031	4857	2488	12,444
Coleoptera	2120	1692	1845	1128	6785
Lepidoptera (moths)	1051	1237	630	741	3659
Apiformes‐Hymenoptera	293	147	852	1687	2979
Syrphidae (Diptera)	1051	378	575	712	2716
Hemiptera (Heteroptera)	291	188	284	204	967
Dermaptera	227	3	2	70	302
Neuroptera	126	43	18	53	240
Orthoptera	36	50	58	12	156
Lepidoptera (butterflies)	38	23	69	15	145
Mecoptera	1	78	21	0	100
Trichoptera	5	6	63	6	80
Blattodea	0	0	0	42	42
Odonata	9	0	10	0	19
Ephemeroptera	0	0	7	3	10
Plecoptera	1	1	3	0	5
Other taxa (group 16)	5500	4315	6345	3634	19,794
Total	171,611	104,795	128,290	128,432	533,128

### 3.1 Biomass‐Abundance Relationship

**FIGURE 2:**
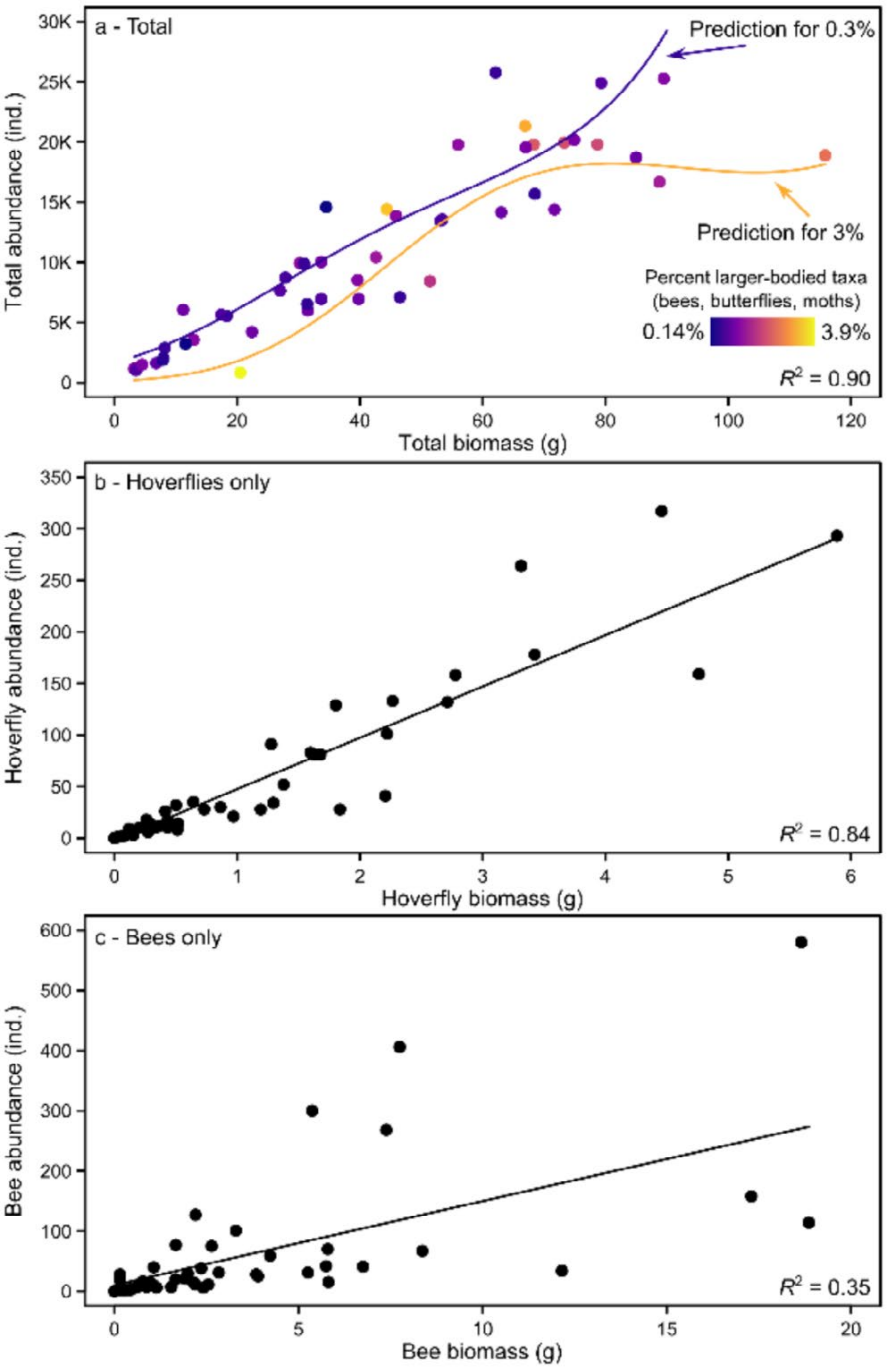
Relationship between total abundance (individuals) and total biomass (g) for (a) the total malaise trap community, (b) hoverflies only, and (c) bees only across all sites, seasons, and sampling years. Each point represents one sample (48 samples in total). Predicted relationships in (a) are shown for communities with less (0.3%; yellow) versus more (3%; purple) larger‐bodied taxa based on a generalized linear mixed model. These example body size values were used to avoid predicting on the boundary of the minimum and maximum body size values (both shown in panel a).

The relationship between biomass and abundance depended on the proportion of larger‐bodied taxa (i.e., bees, butterflies, and moths; Table 1), evidenced by significant interactions between this term and both the linear and quadratic terms for biomass (*Φ* = 1.1; Linear: LRT, *n* = 48, L = 7.0, df = 1, *p* = 0.0082; Quadratic: LRT, *n* = 48, *L* = 4.6, df = 1, *p* = 0.032). For example, communities in which total abundance was comprised of around 0.3% larger‐bodied taxa exhibited an approximately linear biomass‐abundance relationship, gaining about 286 individuals per gram of biomass (Figure 2a). In contrast, communities with an order of magnitude more larger‐bodied taxa (3%) exhibited a non‐linear biomass‐abundance relationship. These communities gained about 258 individuals per gram of biomass until biomass was around 70 g, after which increasing biomass did not translate to increased abundance (Figure 2a). These results were supported by our separate analyses of hoverflies and bees. The biomass‐abundance relationship was more consistent and thus predictable for hoverflies (*R* ^2^ = 0.82; Figure 2b) versus less consistent for bees (*R* ^2^ = 0.35; Figure 2c), indicating a weaker abundance‐biomass relationship within the latter larger‐bodied taxonomic group.

### 3.2 Insect Communities From Malaise Traps

**FIGURE 3:**
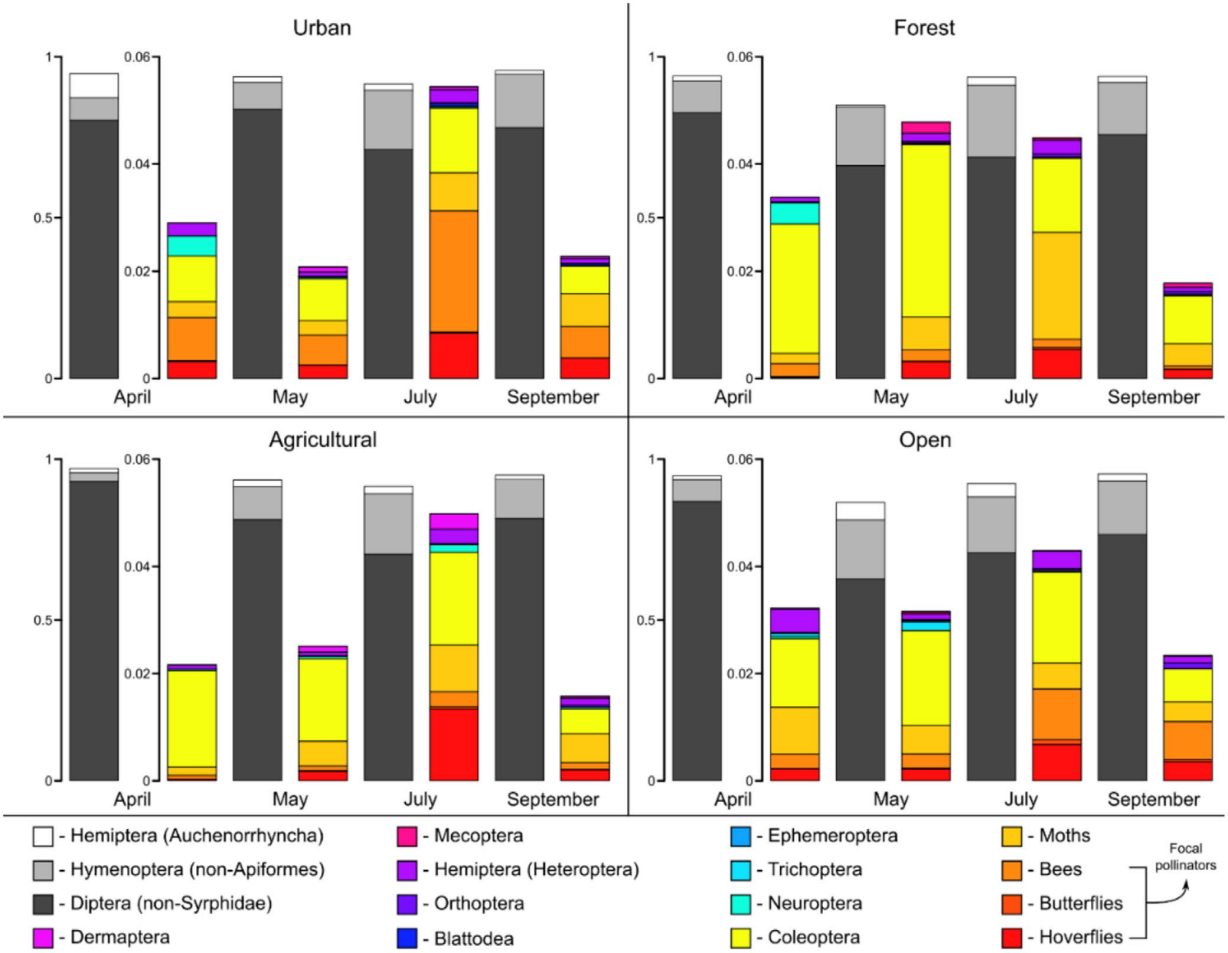
Proportion of each Malaise trap community comprised of each insect taxonomic group across sites and seasons averaged across 2019, 2020, and 2021. Note two different *y*‐axis scales are used to visualize the proportion of dominant (0%–100%) and rare taxa (0%–6%) in the same plot. Additionally, orders Plecoptera and Odonata are present in some samples, but never comprise enough of the community to be visible and so are not shown.

Diptera were consistently the most abundant order across sites and seasons, averaging 77% of total abundance across samples, 99% of which were not hoverflies (Figure 3). Non‐Apiformes Hymenoptera (averaging 14%) and Hemiptera (Auchenorrhyncha; averaging 2%) were the next most consistently abundant. All other taxonomic groups sometimes comprised up to 6% of the community depending on the site and season, especially Coleoptera, but typically comprised < 1% (Figure 3). Focal pollinators always comprised a small proportion of each community, ranging from 0.1% to 3%.

### 3.3 Variation Explained by Land Use, Weather/Climate, and Flowering Plants

**FIGURE 4:**
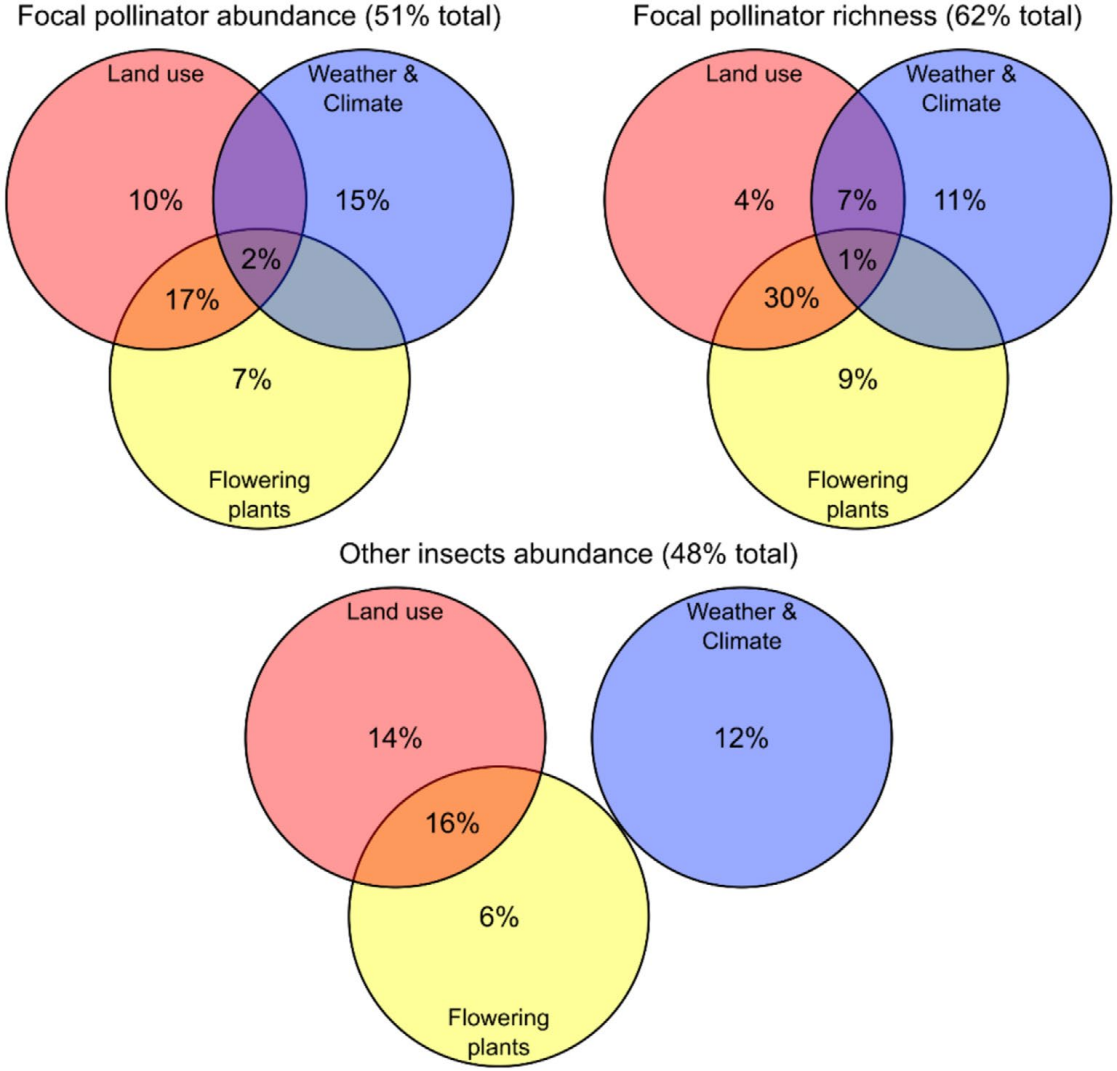
Venn diagrams of the percent of total variance explained by land use, weather/climate, and flowering plants for focal pollinator abundance, focal pollinator richness, and the abundance of other insects. Percentages are derived from the associated variation partitioning analyses.

Based on variation partitioning, land use, weather/climate, and flowering plants explained 51% of total variance in the abundances of focal pollinator families, 62% of variation in pollinator family‐level species richness, and 48% of variation in the abundances of other insects (Figure 4). The individual effects of weather/climate (11%–15%) and the covarying effect of land use and flowering plants (16%–30%) were the primary drivers of these relationships (Figure 4). The individual effects of land use (4%–14%) and flowering plants (6%–9%) were of lesser importance, with little to no influence of the covarying effect of land use and weather/climate (0%–7%), weather/climate and flowering plants (always 0%), nor of covariation among all three predictor groups (0%–2%; Figure 4).

### 3.4 Relationships to Individual Drivers

**FIGURE 5:**
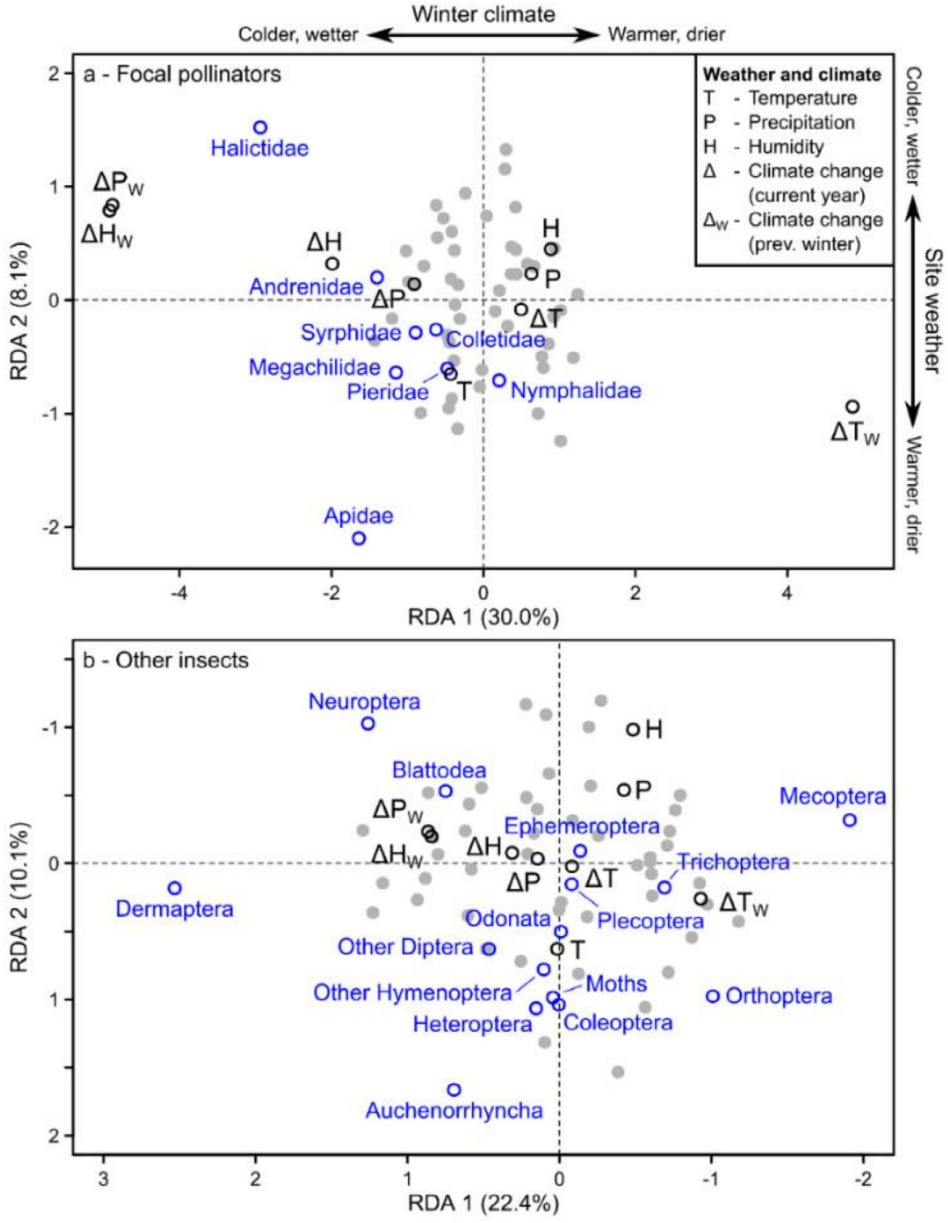
Redundancy Analysis (RDA) of the abundance of (a) each focal pollinator family (blue) and (b) each group of other insects (blue) in relation to weather & climate (black) for all sites, seasons, and years (gray points indicate positions of individual community samples).

**FIGURE 6:**
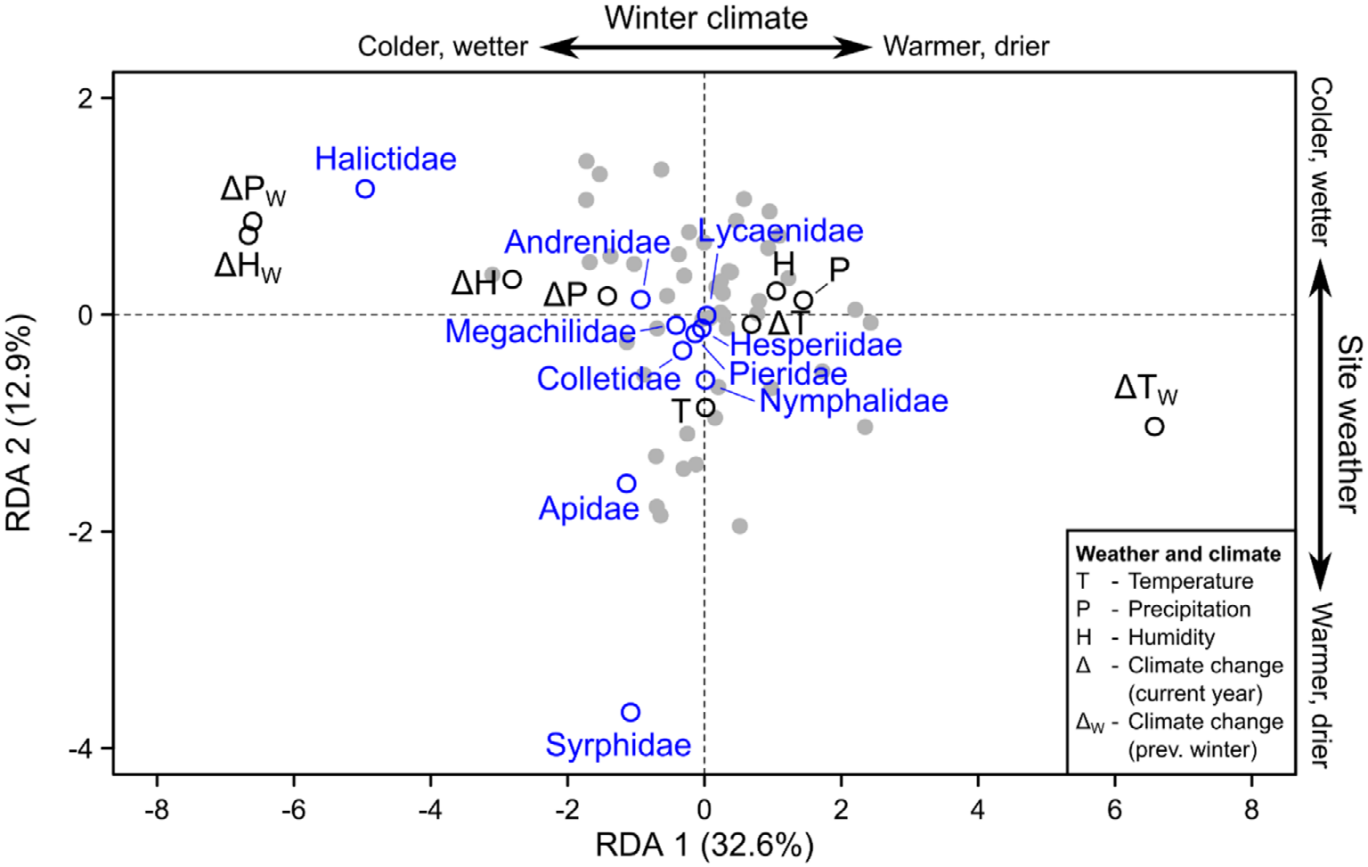
Redundancy Analysis (RDA) of the richness of each focal pollinator family (blue) in relation to weather & climate (black) for all sites, seasons, and years (gray points indicate positions of individual community samples).

We first report insect relationships to weather/climate, given these results are the simplest to interpret because there is little covariation with land use or flowering plants. Based on the weather/climate abundance RDAs, most taxonomic groups, particularly bees (such as Halictidae), Auchenorrhyncha, and Dermaptera, exhibited higher abundances in the habitats that exhibited either warmer and drier weather (lower sides of Figure 5a,b), or that experienced colder and wetter climate anomalies in both the sampling year and the previous winter (left sides of Figure 5a,b). We found the same relationships for focal pollinator richness (Figure 6). In contrast, Mecoptera and somewhat Trichoptera were most abundant in the opposite weather and climate conditions (upper and right sides of Figure 5b).

**FIGURE 7:**
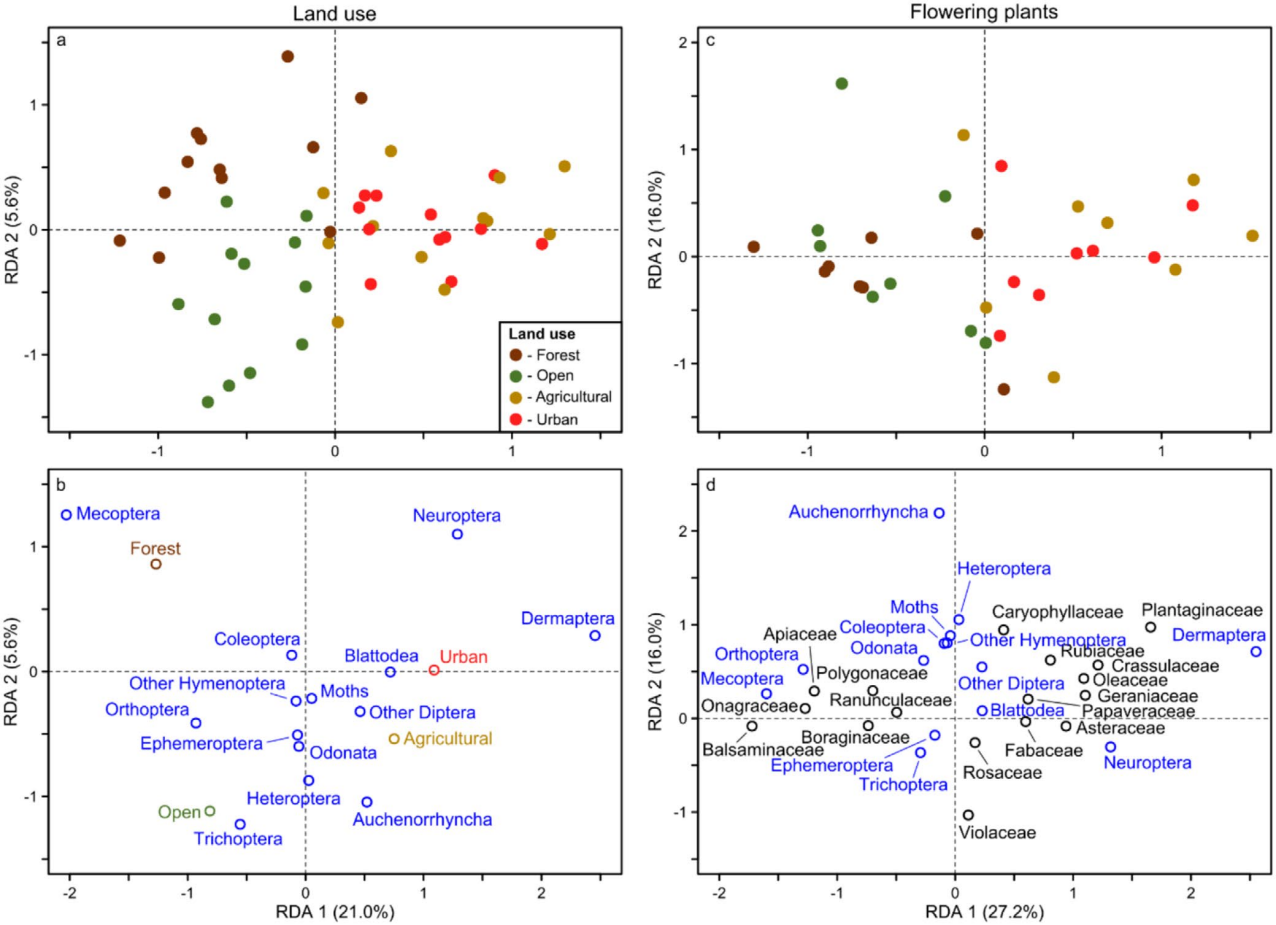
Redundancy Analysis (RDA) of the abundance of each group of other insects (blue) in relation to (a, b) land use and (c, d) flowering plants for all sites, seasons, and years. Colored points indicate the ordination positions of individual community samples (brown = samples from the site dominated by forest cover; green = open land; gold = agricultural; red = urban).

**FIGURE 8:**
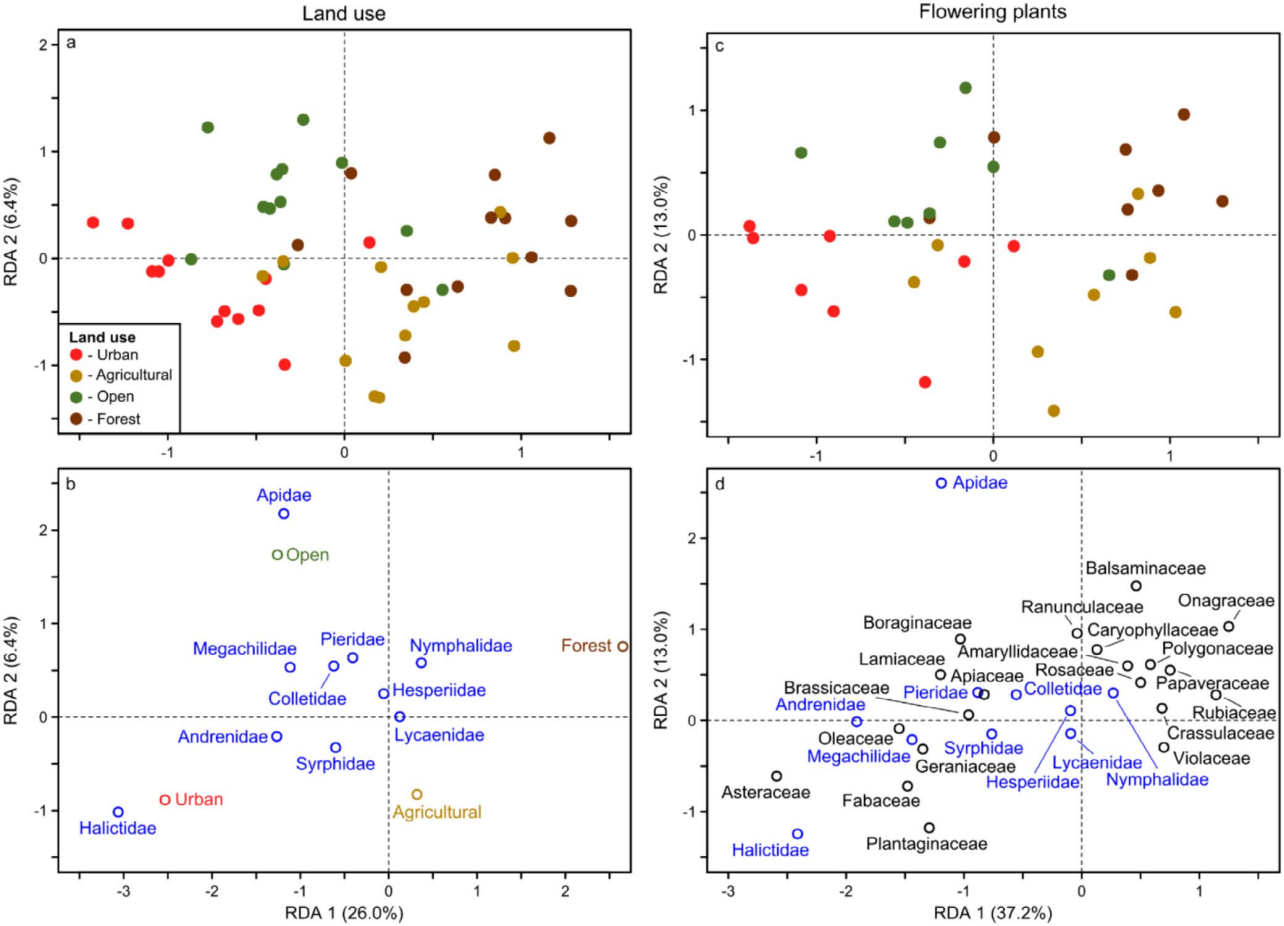
Redundancy Analysis (RDA) of the abundance of each focal pollinator family (blue) in relation to (a, b) land use and (c, d) flowering plants for all sites, seasons, and years. Colored points indicate the ordination positions of individual community samples (brown = samples from the site dominated by forest cover; green = open land; gold = agricultural; red = urban).

**FIGURE 9:**
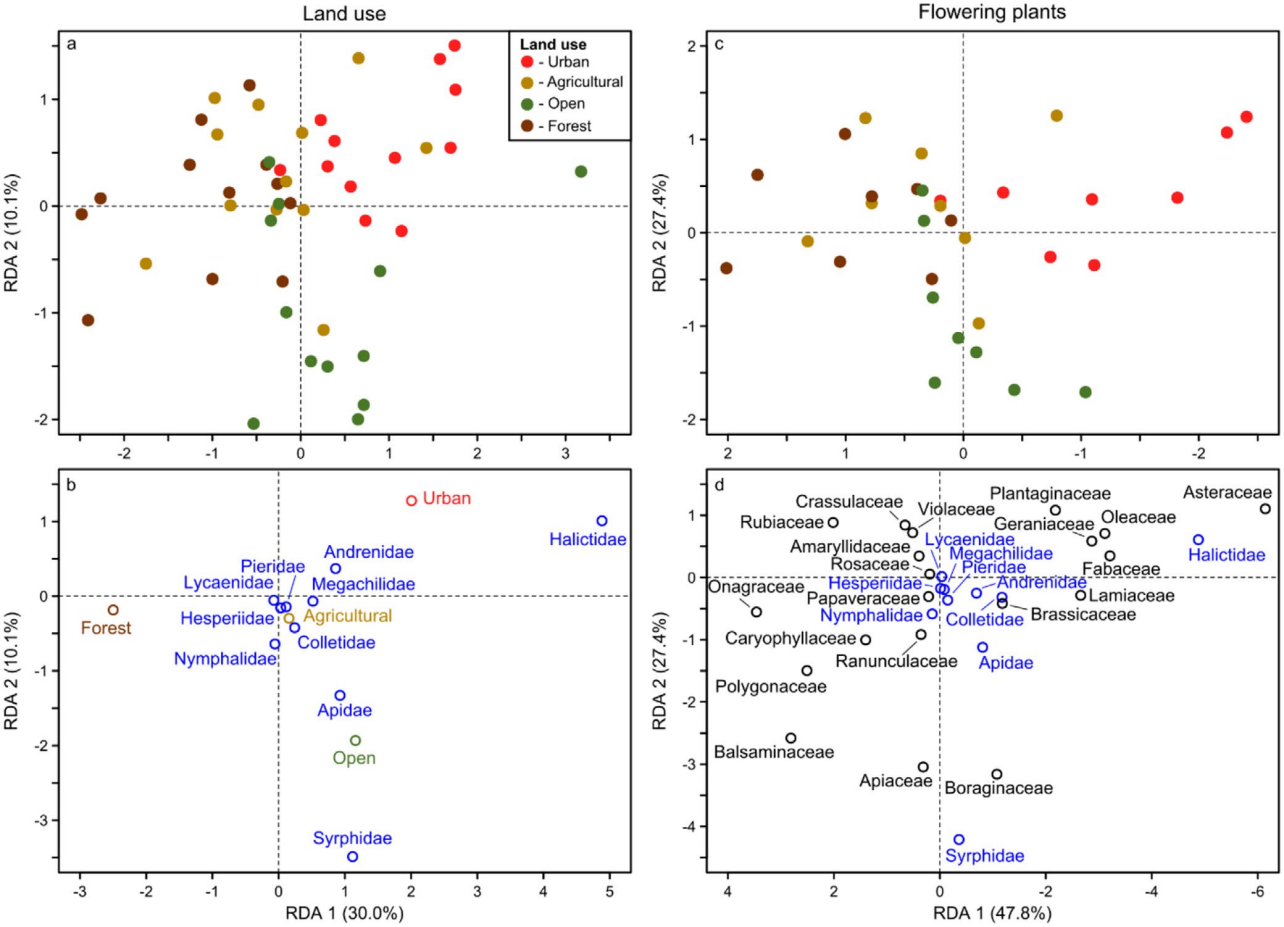
Redundancy Analysis (RDA) of the richness of each focal pollinator family (blue) in relation to (a, b) land use and (c, d) flowering plants for all sites, seasons, and years. Colored points indicate the ordination positions of individual community samples (brown = samples from the site dominated by forest cover; green = open land; gold = agricultural; red = urban).

Regarding land use and flowering plants, covariation between these predictor groups occurred because insects found in certain habitat types were also somewhat related to differences in flowering plants among these habitats. For example, based on the abundance RDA for other insects, the compositional differences explained by land cover were primarily driven by differences between the forested/open land habitats versus the agricultural/urban habitats (i.e., left versus right sides of Figure 7a,b). Specifically, the forested site was characterized by Mecoptera (left side of Figure 7a,b), with more Orthoptera and Trichoptera in the open area site, whereas the urban and agricultural sites were dominated by Dermaptera and Neuroptera (right side of Figure 7a,b). Focal pollinator composition also varied with land cover in a similar manner as the other insects, although the principal differences were between the forested versus urban site, with little to no compositional differences between the open and agricultural sites. The forested site was characterized by generally fewer pollinators overall, whereas more pollinators occurred in the urban site, particularly bees such as Apidae, Andrenidae, and Halictidae (Figures 8 and 9).

We found similar differences between the forested versus urban site in the flowering plant RDAs. For example, Mecoptera in the forested site were primarily associated with Balsaminaceae, Onagraceae, and Apiaceae (left side of Figure 7c,d), whereas Dermaptera and Neuroptera in the urban site were primarily associated with several different plant families, particularly Plantaginaceae and Asteraceae (right side of Figure 7c,d). The focal pollinators exhibited similar relationships. For example, the forested site exhibited lower abundance and richness of most species and was primarily characterized by Balsaminaceae and Onagraceae (Figures 8 and 9). In contrast, the urban site was characterized by different species and flowering plants, primarily Halictidae and somewhat Andrenidae in association with several plant families, especially Asteraceae.

## 4 Discussion

### 4.1 Biomass‐Abundance Relationship

We found a generally strong relationship between total biomass and total abundance, indicating that declines in Malaise‐trap biomass can indicate equivalent declines in the total number of insects. However, this relationship weakened in a subset of communities with a higher share of larger‐bodied species, such as those with larger bees and butterflies (e.g., *Bombus* spp. or *Pieris* spp. in our samples). These results suggested that, in such communities, changes in total biomass may be disconnected from changes in abundance because biomass can strongly decline from the loss of just a few larger‐bodied individuals. The weakening of the biomass‐abundance relationship occurred when biomass reached around 70–80 g after 14 days, which equates to 5–5.7 g per day.

Communities with biomass values in this range (or higher) were more common in previous decades (see Hallmann et al. 2017), but are now rarer due to losses of insect biodiversity, which negatively impacts food webs and ecosystem services (e.g., pollination, decomposition, and pest control) (Lose and Vaughan 2006; Cardoso et al. 2020; Klein et al. 2007; Hof and Bright 2010; Newton 2017; Fartmann 2021). This lower biomass in recent years is supported by other Malaise trap studies from Germany, which indicate that sample biomass rarely reaches above 5 g per day (Hallmann et al. 2017; Uhler et al. 2021; Sinclair et al. 2024). Many Malaise trap communities may therefore now fall within the biomass range where changes in biomass reliably indicate equivalent changes in abundance. However, further verification of a reliable abundance‐biomass relationship at lower total biomass is needed from other European and non‐European regions, as is verification that the abundance‐biomass relationship weakens in communities comprised of more larger‐bodied taxa (e.g., communities with biomass > 6 g per day).

### 4.2 Effects of Land Use, Weather/Climate, and Flowering Plants

Despite focal pollinators (i.e., bees, butterflies, and hoverflies) comprising only a small fraction of the total Malaise trap insect community, we still found that variation in their composition was often explained by similar differences in weather/climate, land use, and flowering plants compared to the other insects. Using weather and climate as an example, variation in the relative abundance and diversity of bees (e.g., Halictidae) and hoverflies was primarily explained by weather temperature and winter climate anomalies for precipitation and humidity, with more taxa in the sites that experienced warmer, drier weather and colder, wetter winters. We found these same general relationships in the other insects. This result is not specific to our study sites because the same relationships have been reported elsewhere in Germany (Müller et al. 2023), attributed to the positive effects of warmer weather during the sampling period on insect abundance and activity, and the benefits of snow during the previous winter for sheltering overwintering insects. However, the role of winter conditions for insect survival may vary among climate zones, particularly with future climate change (Bale and Hayward 2010).

Similarly, although we expected stronger explanatory power of flowering plants for focal pollinator composition, we instead found that plants explained approximately equal variation for both pollinators and other taxa. This likely occurred because many ‘other insects’ are also pollinators, or at least depend on plants as herbivores. For example, pollinators can include moths (van Zandt et al. 2020), beetles (Wardhaugh 2015), and other families of Diptera (e.g., Bibionidae, Stratiomyidae, Tachinidae, Calliphoridae, Conopidae, Bombyliidae, Acroceridae; Rader et al. 2011, 2020; Wardhaugh 2015; Orford et al. 2015) and Hymenoptera (e.g., Tenthredinidae, Vespidae, and Formicidae; Gómez and Zamora 1992; Rader et al. 2020; Labandeira et al. 2007). Potential pollinators from these groups generally receive less attention because they are highly diverse, hard to identify, and their exact role in pollination can be difficult to verify, but they may help explain why flowering plants captured similar variation in other insects as they did for typical bee, butterfly, and hoverfly pollinators.

While the broad driver groups always explained similar compositional variation among taxonomic groups, the specific taxa responsible suggested these relationships were often caused by different mechanisms, particularly regarding land cover and flowering plants. For example, differences between the forested and urban sites, and their associated flowering plants, explained substantial compositional variation in both focal pollinators and other insects. Differences in focal pollinators between these habitats were driven by the Halictidae, which were primarily associated with Asteraceae in the urban habitat. This relationship was likely causally related to the widely distributed Asteraceae, which tend to occur or are purposefully planted more frequently in urban areas, and are used as pollen and nectar sources by the polylectic Halictidae (Amiet and Krebs 2019; Mani and Saravanan 1999). In contrast, compositional differences between the forested and urban sites in the other insects were driven by comparatively rare taxa, specifically Mecoptera in the forest habitat, which were associated primarily with Balsaminaceae ( *Impatiens parviflora* DC. in our forested study site), and Dermaptera in the urban habitat, which were associated with Plantaginaceae. These relationships are likely not driven by these specific flowering plants because neither Mecoptera nor Dermaptera are known to rely on them. Instead, the Mecoptera in our study region (primarily *Panorpa* spp.) are carnivorous (Sauer and Hensle 1977; Byers and Thornhill 1983) and the Dermaptera (primarily *Forficula auricularia* Linnaeus, 1758) are omnivorous, consuming plant parts, fruits, seeds, invertebrates, detritus, etc. (Strenger 1950; Mueller et al. 1988). The relationships to flowering plants in these groups are therefore likely spurious and occur due to covariation with other, more important factors that vary between the forest and urban habitats (e.g., moisture and shade; Byers and Thornhill 1983). Consequently, although these habitat differences explained similar overall variation among insect groups, each group was likely responding to different, underlying mechanisms.

In addition to potentially differing mechanisms between the focal pollinators and other insects, we also found some differing responses to individual habitat characteristics. For example, both our forested and agricultural site exhibited an overall low diversity of focal pollinators, which could be partly due to a lower availability of flowering plants beyond the 100 m radius used in our surveys, such as in more heavily forested areas or nearby wind‐pollinated crops like maize and cereals (Düll and Kutzelnigg 2011). However, when considering other insects, the agricultural site harbored a wide variety of taxa and was more similar to the urban habitat. These differences indicated that the effect of agriculture depended on the insect taxonomic group in question, with the agricultural site being potentially less suitable for certain focal pollinators (e.g., Apidae; Figure 8) versus more suitable for other insects. This differing effect of agriculture between taxonomic groups contrasts with the fact that both exhibited generally lower diversity in the forest habitat and higher diversity in the urban habitat. Consequently, although focal pollinators and other insects responded similarly to certain land cover drivers (or weather/climate drivers as discussed above), their responses to other drivers were highly variable.

## 5 Conclusions

Malaise traps are becoming a principal tool for global insect monitoring (Srivathsan et al. 2023; Seymour et al. 2024). Results from these traps also form a key evidence base for global insect biodiversity loss (Hallmann et al. 2017), and insect responses to land cover and climate change (Uhler et al. 2021; Ganuza et al. 2022; Sinclair et al. 2024). However, questions remain about the degree to which findings from Malaise traps can be extrapolated to different components of the flying insect community. Our results indicate that changes in total Malaise trap biomass generally reflect changes in the total abundance of trapped insects, although this relationship potentially breaks down in certain communities characterized by a higher proportion of larger‐bodied taxa. Additionally, research identifying factors that explain variation in the majority of the insect community (e.g., ‘other insects’ in our study) may be able to infer these same drivers affect other important taxa that comprise a small minority, such as focal pollinators. Such inferences must, however, be made with caution because different taxonomic groups may vary with the same habitat characteristics, like changes in land cover, weather/climate, or flowering plants, but our results suggest the mechanisms underlying this variation can differ widely among taxa. In some cases, certain relationships may be driven by covarying, unmeasured environmental factors. Therefore, findings from Malaise trap studies may apply generally across different insect taxa and to multiple community components; however, care should be taken when using these findings to inform conservation and management to ensure the mechanisms responsible are being addressed.

## Author Contributions

**Nicole Remmel:** conceptualization (equal), data curation (lead), investigation (lead), methodology (lead), writing – original draft (equal), writing – review and editing (lead). **Julian Enss:** data curation (supporting), investigation (supporting), writing – review and editing (supporting). **Peter Haase:** conceptualization (supporting), funding acquisition (lead), supervision (equal), writing – review and editing (supporting). **James S. Sinclair:** conceptualization (equal), formal analysis (lead), supervision (equal), visualization (lead), writing – original draft (equal), writing – review and editing (supporting).

## Conflicts of Interest

The authors declare no conflicts of interest.

## Supporting information


**Data S1:** ece372006‐sup‐0001‐DataS1.xlsx.

**Table S2:** ece372006‐sup‐0002‐TableS1.docx.

## Data Availability

All data and code required to repeat our analyses are privately available for review from https://figshare.com/s/dcb20aba15d94f4691f1. These data will be made publicly available upon acceptance for publication; this statement will be amended at that time.
