# Supplementary material for: Are Findings of Key Insect Metrics Generalizable Across Different Taxa in Malaise Trap Samples?
Source: Ecol Evol. 2025 Aug 15;15(8):e72006. doi: 10.1002/ece3.72006 (PMC12355040; doi:10.1002/ece3.72006)
Supplement: Supplementary file 2 — Table S2: ece372006‐sup‐0002‐TableS1.docx. [file ECE3-15-e72006-s002.docx]

# **Supplementary material**

**Table S1.** Identification keys and references of trait information

| **Taxa group** | **Identification keys** | **References trait information (Body size)** |
| --- | --- | --- |
| Hymenoptera – Apiformes | Amiet *et al.*, 2001, 2004, 2007, 2010, 2014 | Amiet *et al.*, 2001, 2004, 2010, 2014; Amiet & Krebs, 2019 |
| Diptera – Syrphidae | Doczkal & Schmid U., 1994; Bot & van de Meutter, 2020; Veen, 2010 | Bot & van de Meutter, 2020; Ball, 2015; Milne, 1980; Vockeroth, 1992 |
| Lepidoptera (Butterflies, moth) | Settele *et al.*, 2015 | Willner, 2017a, 2017b |

**References**

Amiet, F., Herrmann, M., Mueller, A. & Neumeyer, R. (2010) Apidae 6*. Fauna Helvetica*, 26. Centre Suisse de Cartographie de la Faune, Neuchâtel.

Amiet, F., Herrmann, M., Müller, A. & Neumeyer, R. (2001) Apidae 3. *Fauna Helvetica*, 6. Centre Suisse de Cartographie de la Faune, Neuchâtel.

Amiet, F., Herrmann, M., Müller, A. & Neumeyer, R. (2004) Apidae 4. *Fauna Helvetica*, 9. Centre Suisse de Cartographie de la Faune, Neuchâtel.

Amiet, F., Herrmann, M., Müller, A. & Neumeyer, R. (2007) Apidae 5*. Fauna Helvetica*, 20. Centre Suisse de Cartographie de la Faune, Neuchâtel.

Amiet, F. & Krebs, A. (2019) Bienen Mitteleuropas: Gattungen, Lebensweise, Beobachtung. Haupt Verlag, Bern.

Amiet, F., M. Mueller & A. Neumeyer, R. (2014) Apidae 2*. Fauna Helvetica*, 4. Centre Suisse de Cartographie de la Faune, Neuchâtel.

Ball, S. (2015) Britain's Hoverflies: A Field Guide - Revised and Updated Second Edition. WILDGuides of Britain and Europe Ser, v.17. Princeton University Press, Princeton.

Bot, S. & van de Meutter, F. (2020) Veldgids zweefvliegen. KNNV Uitgeverij, Zeist.

Doczkal, D. & Schmid U. (1994) Drei neue Arten der Gattung Epistrophe (Diptera: Syrphidae), mit einem Bestimmungsschlüssel für die deutschen Arten. *Stuttgarter Beiträge für Naturkunde* (507), 1–32.

Milne, L.J. (1980) The Audubon Society field guide to North American insects and spiders: Lorus and Margery Milne; visual key by Susan Rayfield. (The Audubon Society field guide series). Knopf distributed by Random House, New York.

Settele, J., Steiner, R., Reinhardt, R., Feldmann, R. & Hermann, G. (2015) Schmetterlinge: Die Tagfalter Deutschlands. Ulmer Naturführer. Ulmer, Stuttgart, Hohenheim.

van Veen, M.P. (2010) Hoverflies of Northwest Europe: Identification Keys to the Syrphidae. BRILL.

Vockeroth, J.R. (1992) The flower flies of the subfamily Syrphinae of Canada, Alaska, and Greenland, Diptera: Syrphidae. *The insects and arachnids of Canada* (Part 18), 456 pp.

Willner, W. (2017a) Alle Tagfalter im Porträt. Taschenlexikon der Schmetterlinge Europas / Wolfgang Willner, Tagfalter. Quelle & Meyer Verlag, Wiebelsheim.

Willner, W. (2017b) Die häufigsten Nachtfalter im Porträt. Taschenlexikon der Schmetterlinge Europas / Wolfgang Willner, Band 2. Quelle & Meyer, Wiebelsheim.
